# Combining Multiple Plant Attributes to Reveal Differences in Community Structure in Two Distant Deserts in Central Asia

**DOI:** 10.3390/plants12183286

**Published:** 2023-09-17

**Authors:** Ye Tao, Xiao-Bing Zhou, Ben-Feng Yin, Liliya Dimeyeva, Jing Zhang, Yong-Xin Zang, Yuan-Ming Zhang

**Affiliations:** 1 State Key Laboratory of Desert and Oasis Ecology, Key Laboratory of Ecological Security and Sustainable Development in Arid Lands, Xinjiang Institute of Ecology and Geography, Chinese Academy of Sciences, Urumqi 830011, China; taoye@ms.xjb.ac.cn (Y.T.); zhouxb@ms.xjb.ac.cn (X.-B.Z.); yinbf@ms.xjb.ac.cn (B.-F.Y.); zhangjing@ms.xjb.ac.cn (J.Z.); zangyongxin@ms.xjb.ac.cn (Y.-X.Z.); 2 Institute of Botany and Phytointroduction, Ministry of Ecology and Natural Resources of the Republic of Kazakhstan, Almaty 050040, Kazakhstan; l.dimeyeva@mail.ru

**Keywords:** desert ecosystem, species composition, functional group, similarity index, environmental factor, community conservation

## Abstract

International interest is growing in biodiversity conservation and sustainable use in drylands. Desert ecosystems across arid Central Asia are severely affected by global change. Understanding the changes in a plant community is an essential prerequisite to revealing the community assembly mechanism, vegetation conservation, and management. The knowledge of large-scale spatial variation in plant community structure in different Central Asian deserts is still limited. In this study, we selected the Taukum (TD, Kazakhstan) and the Gurbantunggut (GD, China) deserts as the research area, with similar latitudes despite being nearly 1000 km apart. Thirteen and 15 sampling plots were set up and thoroughly investigated. The differences in community structure depending on multiple plant attributes (individual level: plant height, canopy diameter, and plant volume, and community level: plant density, total cover, and total volume) were systematically studied. TD had a better overall environmental status than GD. A total of 113 species were found, with 68 and 74 in TD and GD, respectively. The number of species and plant attributes was unequally distributed across different families and functional groups between deserts. The values of several plant attributes, such as ephemerals, annuals, dicotyledons, and shrubs with assimilative branches in GD, were significantly lower than those in TD. The Motyka indices of six plant attributes (26.18–38.61%) were higher between the two deserts than the species similarity index (20.4%), indicating a more robust convergence for plant functional attributes. The community structures in the two deserts represented by different plant attribute matrices demonstrated irregular differentiation patterns in ordination diagrams. The most variance in community structure was attributed to soil and climatic factors, while geographic factors had the smallest proportion. Consequently, the community structures of the two distant deserts were both different and similar to an extent. This resulted from the long-term impacts of heterogeneous environments within the same region. Our knowledge is further deepened by understanding the variation in community structure in different deserts on a large spatial scale. This therefore provides valuable insights into conserving regional biodiversity in Central Asia.

## 1. Introduction

Biological resources combine genes, species, and ecosystems with realistic and potential values for human beings. They are the essential components of biological diversity necessary for human survival and development [1,2,3]. Abundant plant germplasm resources differentiated from non-desert areas have been bred through long-term adaptation and evolution [4,5]. These plant resources indicate significant social, economic, and ecological values and are important for humankind [5,6]. Desert ecosystems in drylands are fragile, and vegetation helps to maintain the stability of the desert ecosystem. Some types of desert ecosystems are even on the verge of collapse due to the impact of climatic and land-use changes. This threatens the survival and diversity of many plant species [3,7]. The stability of the desert plant community is the expression of various important functions and has positive significance for the sustainable development of desert ecosystem [8]. The 15th Meeting of the UN Conference on Biological Diversity (known as COP15) noted that biodiversity conservation and sustainable use policies in drylands (including desert areas) are relatively lacking, and the unique biodiversity has not received enough attention. It is critical to strengthen international cooperation and prioritize biodiversity conservation in drylands (https://www.unep.org/un-biodiversity-conference-cop-15, accessed on 31 January 2023). Hence, the comparative study of the composition and structure of different desert plant communities sheds light on the community assembly mechanism, forecasting future dynamics and guiding biodiversity conservation and scientific management [4,9].

Identifying patterns in community structure is essential in plant ecology, as plant community structure facilitates the measurement of ecosystem function and ecological services [10,11,12]. However, no standard approach exists for describing or comparing community structures [10]. The functional groups can reflect the combination of species depending on functional responses to environmental variables or ecological processes, irrespective of taxonomic affiliation [13,14,15]. Therefore, species are allocated to functional groups according to particular objectives, e.g., phylogeny, life form, photosynthetic pathway, woody/herbaceous, grass/non-grass, legume/non-legume, or even taxon [10]. However, it is not easy to comprehensively explore the plant community structure if the presence or absence of binary data only represents the species in a community. The plant body size can reflect the status and role of species in the community, expressed by plant attributes, including canopy diameter, plant height, canopy cover, plant volume, and biomass [16,17,18,19]. For instance, a recent study has indicated that canopy occupation volume can effectively quantify the photosynthetic capacity of the plant canopy. Moreover, it also reflects the ability of plants to occupy space resources. Canopy occupation volume describes most photosynthetic changes compared with the canopy cover [20]. Moreover, plant volume is also an excellent biomass prediction parameter, closely associated with vegetation productivity [21]. Thus, combining multiple plant attributes (e.g., cover, height, canopy diameter, and volume) can deeply reveal the differences in plant community structures (i.e., functional structures).

In recent decades, precipitation has exhibited spatially heterogeneous changes in Central Asia [22]. Additionally, the temperature has increased rapidly to approximately twice that of the global average [23]. Several case studies have assessed the effects of biotic and abiotic factors on vegetation dynamics [24,25], net primary productivity, community structure, and species composition [26] across several deserts of Central Asia. Reports have indicated that the vegetation greenness of desert shrubs and sparse vegetation was significantly reduced on the regional scale. They are susceptible to short-term climatic variations [25]. Rather than edaphic heterogeneity, precipitation and topographic heterogeneity are more closely correlated to species richness and distributions in temperate deserts on the local scale. However, different microtopographic conditions shape different plant communities and soil properties on a sand-dune scale [27,28]. Studies comparing the effects of multi-environmental factors on community structures across different deserts in Central Asia under global change have not adequately undertaken.

The current study chose two distant temperate deserts in Central Asia (Taukum Desert and Gurbantunggut Desert, abbreviated as TD and GD, respectively) as the research area. They are nearly 1000 km apart and have similar latitudes. According to existing studies [29,30], the plant functional groups of the two deserts are similar to some extent (ephemerals, ephemeroids, and shrubs with/without assimilative branches, for example). However, the environmental context differs noticeably [4]. Is there a difference in plant community structure between the two deserts? And what are the motivators? Given the vast differences in environmental conditions between two deserts, we hypothesize that significant differences in plant community structure exist, which are primarily influenced by climatic and soil factors. Our goal was to discover the reasons for the heterogeneity of plant community structure in different deserts. Furthermore, important insights into biodiversity conservation and management in arid Central Asia have been provided.

## 2. Results

### 2.1. Differences in Plant Species and Functional Groups

**Figure 1:**
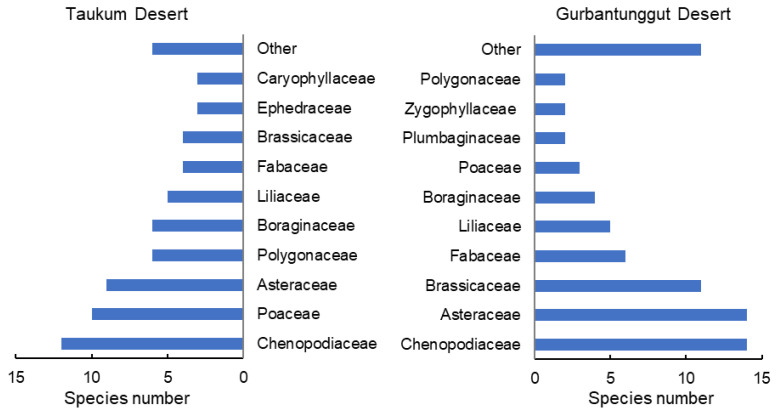
Number of species of different families in plant communities in the Taukum Desert and the Gurbantunggut Desert in Central Asia.

Sixty-eight species belonging to 47 genera and 16 families were identified in TD. Comparatively, there were 74 species from 60 genera and 21 families in GD (Figure 1). In the two deserts, 113 species were recorded. The two desert plants’ dominant families and single-species families were not identical. Dicotyledons, ephemerals, and ephemeroids were more abundant in GD. TD also had more monocotyledons, perennials, and shrubs, particularly shrubs with assimilative branches (Figure S1). Therefore, the plant species composition at the family and functional group levels both markedly differed between the two deserts.

### 2.2. Plant Attributes of Different Functional Groups

Plant density, total cover, and volume exhibited no significant differences between TD and GD for all community species (Figure S2). However, they were higher in TD than in GD, especially for plant density (3.20 ± 1.18 × 10^4^ plants plot^−1^ vs. 1.52 ± 0.36 × 10^4^ plants plot^−1^) in terms of average values. In TD, Chenopodiaceae and Cyperaceae had the highest density for different families (46.72% and 32.95%, respectively). The dominant species were *Ceratocarpus arenarius* and *Carex physodes* (78.84%). In contrast, *C. physodes* (Cyperaceae) demonstrated the highest density (68.77%) in GD (Figure S3). Different families also indicated a clear distinction between deserts.

**Figure 2:**
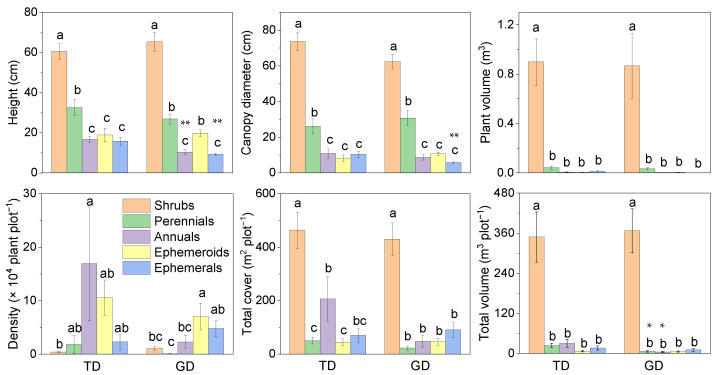
Plant characteristics of different life forms differ in plant communities in the Taukum Desert (TD) and the Gurbantunggut Desert (GD) in Central Asia. Different lowercase letters indicate significant (*p* < 0.05) differences between the different groups in each desert; * and ** indicate significant differences between the two deserts at *p* < 0.05 and *p* < 0.01, respectively.

Annual and ephemeral plant height was greater in TD than in GD (Figure 2). The variation pattern of canopy diameter was the same for the two desert plants among the five life forms. The canopy diameter of ephemerals in GD was significantly lower than in TD. There was a consistent changing trend among the individual plant volumes of the five life forms between the two deserts. The trend of plant density was different from the other five attributes. In TD, the plant density was dominated by annuals (mainly *C. arenarius*), followed by ephemeroids (*C. physodes*), and shrubs were the lowest. Comparatively, ephemeroids dominated the plant density in GD (*C. physodes*), followed by ephemerals, with the lowest density among perennials. The total plant cover did not differ significantly between deserts for the five life forms, and the total plant volume indicated a similar trend in individual plant volume. However, the total plant volume of perennials and annuals in GD was significantly lower than in TD.

**Figure 3:**
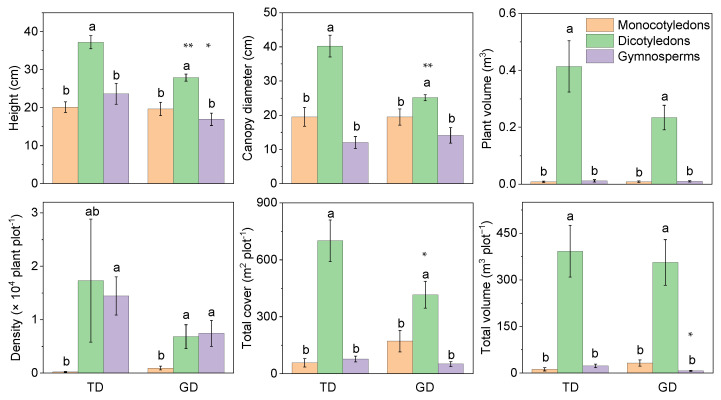
Differences in plant characteristics of different phylogenetic types in plant communities in the Taukum Desert (TD) and the Gurbantunggut Desert (GD) in Central Asia. Different lowercase letters indicate significant (*p* < 0.05) differences between the different groups in each desert; * and ** indicate significant differences between the two deserts at *p* < 0.05 and *p* < 0.01, respectively.

The plant cover, volume, and individual attributes showed that the dicotyledons were significantly higher than the gymnosperms and monocotyledons in TD and GD for different phylogenetic groups (Figure 3). However, plant attributes of some groups between the two deserts differed significantly, e.g., the plant height of monocotyledons and dicotyledons, and the canopy diameter and total cover of dicotyledons. The total volume of monocotyledons was significantly lower in GD than in TD. Dicotyledons had lower individual plant volume in GD than in TD, despite there being no other significant difference between them. The changing trend of plant density differed from the other five attributes. Gymnosperms in both deserts were much lower than dicotyledons and monocotyledons, without any significant difference between the latter two.

**Figure 4:**
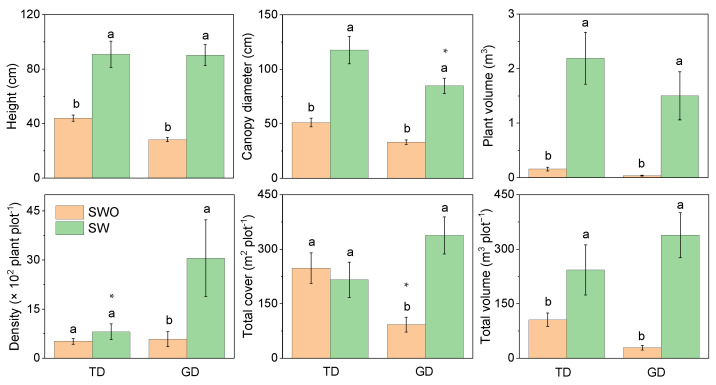
Differences in plant characteristics of shrubs with (SW) and without assimilative branch (SWO) in plant communities in the Taukum Desert (TD) and the Gurbantunggut Desert (GD) in Central Asia. Different lowercase letters indicate significant (*p* < 0.05) differences between the different groups in each desert; * indicate significant differences between the two deserts at *p* < 0.05 and, respectively.

In the two deserts, the plant height, canopy diameter, individual plant volume, and total plant volume of shrubs without assimilative branches were significantly higher than for those with assimilative ones (Figure 4). However, the canopy diameter of shrubs with assimilative branches in GD was substantially lower than in TD. This was because of the large proportion of *E. przewalskii* in GD. No significant difference in plant density between the two types of shrubs in TD was observed. However, the shrub density in GD was significantly higher with assimilative branches than without. The total cover also demonstrated the same trend. However, the total cover of shrubs without assimilative branches in GD was lower than in TD.

### 2.3. Similarity of Species Composition and Plant Attributes for Different Functional Groups

**Figure 5:**
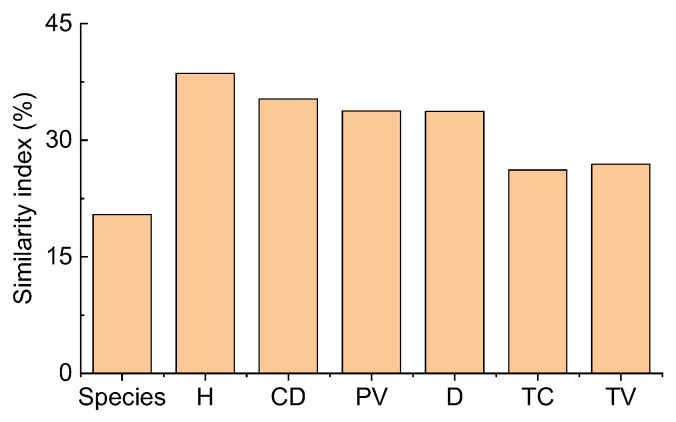
Similarity indices of Sørensen (species) and Motyka in plant communities based on six plant attributes between the Taukum Desert and the Gurbantunggut Desert in Central Asia. H: plant height; CD: canopy diameter; PV: plant volume; D: density; TC: total cover; TV: total volume.

Comparing the two deserts, the Sørensen similarity index of all species was only 20.4%. It was lower than the Motyka indices of six plant attributes for all species (26.18–38.61%) (Figure 5). The Motyka indices of six plant attributes (except for plant height for shrubs) of shrubs, perennials, annuals, and ephemerals were lower than the Sørensen similarity index for different life forms. In contrast, ephemeroids (except plant height) had the opposite trend. Thus, the Motyka indices of most plant attributes of ephemeroids were higher than other life forms (Figure S4). In contrast, perennials exhibited relatively lower Sørensen and Motyka indices than others. The plant density of perennials had the lowest Motyka index (0.23%), and the Motyka index (92.84%) was the highest for the density of ephemeroids. For dicotyledons, the Sørensen index was 40.0%, higher than the Motyka indices of all six plant attributes (16.63–39.51%). However, the Motyka indices (20.83–42.39%) of the other five plant attributes were lower than the Sørensen index except for the Motyka index (50.78%) of canopy diameter for monocotyledons which was higher than the Sørensen index (42.86%) (Figure S5). The Motyka indices (4.92–23.80%) of the other five plant attributes were lower than the Sørensen index (31.58%) except for the plant density (41.23%) of shrubs without assimilative branches. For shrubs with assimilative branches, the Sørensen (62.5%) and Motyka (63.29%) indices of plant height were higher than the Motyka indices (18.64–53.33%) of the others, and plant density had the lowest value (18.64%) (Figure S5). Consequently, depending on different plant attributes between TD and GD, the species and structure similarity differed obviously. Various functional groups and plant attributes represented differential similarity patterns.

### 2.4. Relationships between Plant Attributes and Environmental Factors

The plant height was significantly associated with 11 of 15 environmental factors, such as longitude, DSR, VPD, SWC, MST, TN, and TK, for all species in the community (Table S1). Canopy diameter was significantly correlated with seven environmental factors. Comparatively, the other four attributes were only significantly associated with one or two environmental factors. Most plant attributes of shrubs, perennials, ephemeroids, and ephemerals were weakly correlated with environmental factors for different life forms (Table S2). In contrast, the plant attributes of annuals were significantly associated with one to six environmental factors. The correlation coefficients between the six attributes of monocotyledons and environmental factors were the lowest for the three phylogenetic groups (Table S3). The other five attributes of gymnosperms were significantly correlated with two to four environmental factors, except for plant height. The canopy diameter, plant height, and total cover of dicotyledons indicated a relatively strong correlation with seven to ten significantly related environmental factors. The plant attributes of shrubs without assimilative branches and environmental factors (except for latitude, aridity, and TS) had much stronger correlations than for those with assimilative branches (Table S4). The longitude, MAP, VPD, SWC, and TN demonstrated significant correlations with all plant attributes of shrubs without assimilative branches. Therefore, there was considerable variation in the relationship between the different plant attributes of different functional groups and environmental factors without any consistent pattern.

### 2.5. Influencing Factors of Plant Community Structure Based on Different Plant Attribute Matrices

**Figure 6:**
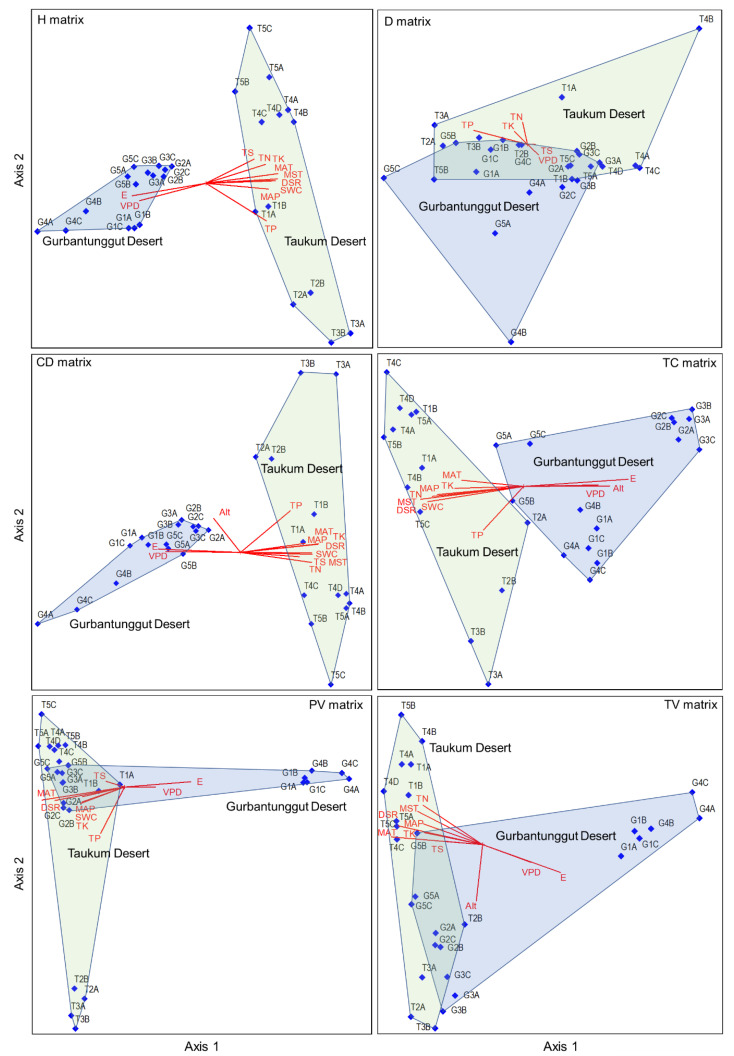
The CCA ordination plots between environmental variables and six plant attribute matrices in plant communities in the Taukum Desert (T) and the Gurbantunggut Desert (G) in Central Asia. H: plant height; CD: canopy diameter; PV: plant volume; D: density; TC: total cover; TV: total volume. E: Longitude; N: Latitude; Alt: Altitude; MAP: Mean annual precipitation; MAT: Mean annual temperature; DSR: Daily solar radiation; VPD: Vapor pressure deficit; SWC: Volumetric soil water content; MST: Mean soil temperature; SOC: Soil organic content; TN: Soil total nitrogen; TP: Soil total phosphorous; TK: Soil total potassium; TS: Total salt content.

The CCA results indicated that longitude, MAP, MAT, DSR, VDP, SWC, MAST, TN, TP, TK, and TS primarily affected plant height and canopy diameter matrices. The longitude and VDP were negatively correlated with Axis 1. Other environmental factors demonstrated an opposite trend (Table S5 and Figure 6). DSR, MAT, and TP mainly affected individual plant volume matrices, among which DSR and MAT were negatively associated with Axis 1 and TP with Axis 2. The plant density was most affected by TP on Axis 1 and TN on Axis 2. Longitude, altitude, MAP, MAT, DSR, VPD, SWC, MST, and TN possessed the highest correlation coefficients with Axis 1 for the total cover matrix, in which longitude and altitude showed a positive and the rest represented a negative correlation. The main factors influencing the total volume matrix were longitude, MAT, DSR, TK, and altitude. Among these, altitude was negatively correlated with Axis 2, and the rest were associated with Axis 1. The influencing factors of the six plant attributes varied, and the sampling plot positions were also not identical in the two deserts in the CCA diagrams. The sampling plots in TD and GD were wholly separated based on plant height, canopy diameter, and total cover matrices (Figure 6). This indicated that the community structures that depended on these three attributes differed significantly between the two deserts. However, the communities in the two deserts could not be separated based on plant density, individual plant volume, and total plant volume matrices.

**Figure 7:**
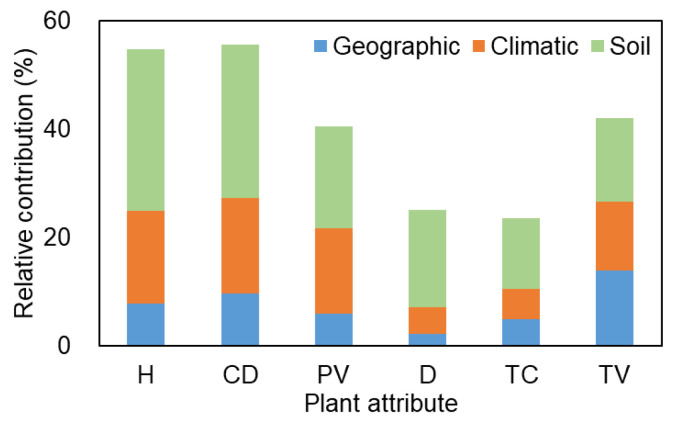
Relative contribution (%) of geographic, climatic, and soil variables to community structure variation based on six plant attributes in two deserts in Central Asia.

In community structures, soil, climatic, and geographic variables jointly revealed more variation (54.8%, 55.6%, and 40.6% for plant height, canopy diameter, and individual plant volume, respectively) based on the three separate plant attribute matrices than community attributes (25.1%, 23.5%, and 42.0% for plant density, total cover, and total volume, respectively) (Figure 7). Soil factors had the most variation in plant attribute matrices, followed by climatic factors, except for total volume (soil > geographic > climatic). By contrast, geographic factors indicated the lowest variation.

## 3. Materials and Methods

### 3.1. Study Area

**Figure 8:**
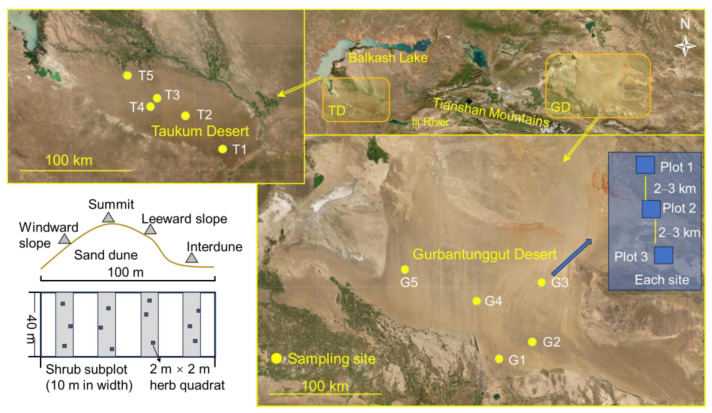
Study area, sampling sites, shrub plots, and herb subplots on sand dunes in the Taukum Desert (TD; T1–5) and Gurbantunggut Desert (GD; G1–5) in Central Asia.

Central Asia covers a large 5 × 10^6^ km^2^ land area, with over 80% of the global temperate deserts. It comprises the vast drylands of north-western China, southwestern Mongolia Republic, and the five Central Asian countries. Globally, central Asia has one of the largest non-zonal arid regions [31]. The study sites are TD in south-eastern Kazakhstan and GD in north-western China. These regions have typical temperate inland deserts from Central Asia. The straight-line distance between the two desert centers is approximately 1000 km (Figure 8), and the climate and soil properties significantly differ (Table 1).

TD is located south of Balkash Lake and the Ili River in the Balkash-Arakol Basin. It extends to the south of the lower course of the Ili River, from the southern end of Lake Balkhash to the Bozoi Plateau. The desert is about 1.0 × 10^4^ km^2^, with a length and width of 240 km and 40–60 km, respectively. The main plant species include *Calligonum leucocladum*, *Ammodendron bifolium*, *Ephedra lomatolepus*, *Bassia prostrata*, *Agropyron fragile*, *Artemisia songarica*, *Krascheninnikovia ceratoides*, and black saxaul (*Haloxylon ammodendron*) [32,33]. Moreover, some places have biological soil crusts (https://dic.academic.ru/dic.nsf/bse/138436/Taykym, accessed on 30 April 2015).

GD is the second largest desert (4.88 × 10^4^ km^2^) in China. It is also the largest fixed/semi-fixed desert in China and is located south of the Junggar Basin between the Tianshan and Altay Mountains. *H. ammodendron*, *H. persicum*, *E. przewalskii*, *A. songarica*, *A. terrae-albae*, and *C. leucocladum* primarily dominates the natural vegetation [30]. There are abundant ephemeral and ephemeroid species, contributing about 40–50% of productivity during spring. Most of the sand surface is covered by biological soil crusts, involving algae, lichen, and moss [34].

### 3.2. Vegetation Sampling

We conducted the study in June–July of 2011–2012 (2011 for TD; 2012 for GD). Five sampling sites were set up in both deserts. Each site was composed of 2–4 sampling plots (i.e., communities) (T1–3 in TD had two plots, and T4 and T5 had four and three plots, respectively; GD had three plots in each site). The plot size was 100 m × 40 m, i.e., the width of the plot was 40 m, and the length covered the whole dune (from the windward slope to the inter-dune area). The plots in a site were separated by 2–3 km. Because surveying the entire 100 m × 40 m plot is difficult, each plot had four 10 m × 40 m subplots for shrubs, and the long side extended in the same direction as the dune. The four subplots were located on the windward slope, leeward slope, summit of the dune, and inter-dune area (Figure 1). The four corners of each plot or subplot were marked with wooden stakes. The herbaceous species were surveyed with three 2 × 2 m areas nested within each subplot. Finally, there were 13 communities (plots) in TD (including a total of 52 shrub subplots and 156 herb quadrats) and 15 in GD (including a total of 60 shrub subplots and 180 herb quadrats).

### 3.3. Species and Functional Group Composition

All the vascular plant species were recorded, and the number of species in a community was defined as the species richness. All the recorded species were classified after the life-form classifications of Zhang and Chen (2002) as either shrubs; perennial herbs with long vegetative periods (perennials); perennial herbs with a short vegetative period (ephemeroids); annuals with a long vegetative period (annuals); or annuals with a short vegetative period (ephemerals) [30]. The phylogenetic types (i.e., gymnosperms, dicotyledons, and monocotyledons) and shrubs with (SW; i.e., without leaves) or without assimilative branches (SWO; i.e., with leaves) were also considered functional groups. Flora of China [35], Flora of Kazakhstan [36], and Flora Xinjiangensis [37] were utilized for nomenclature based on the Engler System.

### 3.4. Plant Attributes

Two types of plant attributes were quantified, i.e., individual and community levels. The individual plant attributes comprised plant height (cm), canopy diameter (cm), and plant volume (m^3^). The plant community attributes involved plant density (plant plot^−1^), total plant cover (m^2^ plot^−1^), and total plant volume (m^3^ plot^−1^). Canopy diameter = (*C_L_* + *C_W_*)/2, where *C_L_* is the canopy length at the widest point, and *C_W_* is the canopy width at the shortest point. The plant volume was estimated as an elliptical cylinder: *C_L_*/2 × *C_W_*/2 × π × height; total plant cover = Σ*C_L_*/2 × *C_W_*/2 × π; and total plant volume = Σ*C_L_*/2 × *C_W_*/2 × π × height [38].

### 3.5. Similarity of Species and Plant Attributes

Based on total species and plant attributes, similarity coefficients of community structure were analyzed using the Sørensen and Motyka indices [39,40,41]. The Sørensen index = 2c/(a + b) × 100, where a and b are the numbers of species from communities *A* and *B*, respectively, and c is the number of species shared by both communities. The Motyka index = 2Σ *M_W_*/(*M_A_* + *M_B_*) × 100, where *M_A_* and *M_B_* are the sums of an attribute of all species from communities *A* and *B*, respectively. *M_W_* is the sum of the corresponding features of the species shared by the communities. The Sørensen and Motyka indices were determined for all species and various functional groups.

### 3.6. Environmental Factors

Soil variables: We collected one soil sample (0–10 cm depth) from each subplot using the five-point method. Thus, four replicates were gained from each community. The total soil samples were 112 (= 28 × 4), the same with the number of shrub subplots. Soil samples were air dried in the shade. Then, a sieve was used to remove the surface organic material and fine roots. Soil organic carbon (SOC, g kg^−1^), total nitrogen (TN, g kg^−1^), total phosphorous (TP, g kg^−1^), total potassium (TK, g kg^−1^), and total salt content (TS, %) were determined in the laboratory based on Bao (2000) [42]. In addition, we obtained soil volumetric water content (SWC, *v*/*v*) and mean soil temperature (MST, °C) from WorldClim version 2.0 (http://worldclim.org/version, accessed on 1 June 2016).

Climatic data: WorldClim version 2.0 (http://worldclim.org/version, accessed on 1 June 2016) was used to retrieve the mean annual precipitation (MAP, mm), mean annual temperature (MAT, °C), daily solar radiation (DSR, Wm^−2^), and vapor pressure deficit (VPD, kPa). We extracted aridity (= 1-aridity index (AI), where AI is the ratio of precipitation to potential evapotranspiration) from the CGIAR-CSI database (http://www.cgiar-csi.org, accessed on 1 November 2018).

Geographic factors: The longitude (E, °), latitude (N, °), and altitude (Alt, m) of each plot center were recorded and utilized as the geographic factors.

The differences in soil and climatic variables are represented in Table 1.

### 3.7. Statistics

The Kolmogorov–Smirnov test was used to check the normality of all the data. The *t*-test (*n* = 2) and one-way ANOVA (*n* > 2) were used to compare the differences in plant attributes between different functional groups in each desert using data (species and functional group composition, species richness, and plant attributes) from the two deserts. Levene’s test was utilized to test for homogeneity, and Duncan’s test was used to perform post hoc multiple comparisons (*α* = 0.05). In addition, when variances were not homogeneous, a robust T2 Tamhane’s test was used [38]. SPSS 19.0 statistical package (SPSS Inc. Chicago, IL, USA) was used for data analyses.

For each functional group, Pearson’s correlation analysis was used to assess the relationships between environmental factors and plant attributes at individual and community levels. The canonical correspondence analysis (CCA) was employed to test the correlations between the environmental variable matrix (28 plots × 15 factors) and total species matrix (with data from each of the six plants attributed to all the species in each community) within the two deserts using PC-ORD version 5 (MjM Software, Oregon) for exploring the community structure–environment relationship [43]. Using a random starting configuration, the Sørenson (Bray–Curtis) distance was used for CCA analysis. A stable two-dimensional solution was finally identified for all six ordinations. In an ordination space, the graphs of plant communities and environmental variables with overlays were used to describe ordination gradients. The environmental gradients represented by the ordination axes were described with Pearson’s correlation to assess significant relationships between each ordination axis and environmental variable. The significance of Pearson’s correlations was determined with the critical values for correlation coefficients. CCA was used to determine the proportion of variance explained by different types of environmental variables [43].

## 4. Discussion

### 4.1. Differences in Plant Species, Attributes, and Community Structure in Desert Areas

We investigated 68 and 74 plant species in TD and GD, respectively. However, the limited investigations did not reflect the actual situation of species within the two deserts. Previous research indicates that populations of 208 species of vascular plants belonging to 30 families and 123 genera from GD were lower than the Kazakhstan deserts [29,30]. GD had a similar life-form composition to TD, having many shrubs with assimilative branches, ephemerals, and ephemeroids. However, GD was dominated by ephemeral plants and followed by shrubs. In contrast, TD displayed the opposite trend. The GD species were concentrated in Chenopodiaceae, Asteraceae, and Brassicaceae, with many monotypic and oligotypic genera species, but this was not the same in TD. These results depicted similar functional group composition, but different species distributions characterized the two deserts (Figure 1). The GD flora is associated with the desert transition zone from east to west in Central Asia [29]. However, some unique floristic elements and plant communities were highlighted in GD, different from the deserts in the east and west of Central Asia [5]. Therefore, the species structures of TD and GD differed significantly.

Attribute-based approaches provide a holistic understanding of the processes driving desert plant communities, linking individual characteristics of organisms affecting performance and community functions [16,19,44,45]. The relationship between plant functional traits (i.e., attributes) and environmental variables indicates the composition of plant communities, defines plant functional groups, and highlights their adaptation to environmental conditions [46]. In the coastal dunes of southern Brazil, using 40 functional characteristics can distinguish the structural differences between woody and herbaceous plant groups and pioneer plant communities. The most significant traits demonstrating plant adaptations to coastal environments include plant height, sclerenchyma, spongy parenchyma, and reserves of inulin in the root, indicating vegetation differentiation in coastal dunes [47]. The projective cover and aboveground biomass represent substantial spatial complexities. Moreover, the projective cover is also an accurate indicator of plant-to-plant competition. Therefore, individual plant numbers, projective cover, and aboveground biomass have indicated different aspects of population or community functions, indicating a better understanding of the structure and function of plant populations. Our results revealed an inconsistent distribution of plant height, density, and total cover among different life forms in TD and GD communities. Although both deserts had shrubs with assimilating branches, they also demonstrated significant differences in crown diameter, density, and the total cover. This reflected the differences in life-form composition characterized by different plant attributes.

The structural differences in plant communities can be well reflected by the similarity indices of species and plant attributes. The Sørensen index and Motyka index can represent them [10]. The Sørensen similarity index comparing both communities was 73% for trees having more than l cm DBH in two abandoned henequen plantations in Mexico. However, the Motyka index for the same individuals depending on the basal area was only 17%. Although the two communities are similar in composition, their biomass distribution is significantly different [40]. Similarly, after 20 years of natural regeneration, a tropical human-modified forest has relatively high similarities to the primary reference forest in the presence and absence of native sapling species (Sørensen similarity index: 38%) but not in terms of quantity (Motyka index: 13%). Meanwhile, there is a higher similarity between two tropical human-modified forests for species than for quantity. Therefore, their compositions converge based on the quality of native saplings species, but not quantity [41]. However, in our study, the Motyka indices of six plant attributes of all species (26.18–38.61%) were higher than the Sørensen similarity index (20.4%), which was different from the studies mentioned above. Low similarity indices indicated that the species and structure similarity differed vastly based on various plant attributes between TD and GD. Different functional groups and plant attributes represented differential similarity patterns. Besides this, although live or dead biomass in three shrub communities differed considerably on the local scale in GD, all the ratios of total dead biomass to total biomass were close to 1/4. Additionally, shrubs accounted for most of the aboveground biomass, while annuals contributed only 5.4–5.7%. These results depicted that the species composition of the three shrub communities within the same area differed to an extent. However, the three communities had some similarities from the perspective of community biomass structure [21]. Consequently, this study had different species structures for TD and GD communities. Contrastingly, the plant attributes (e.g., functional traits) exhibited relatively high similarity (although the Motyka index was not more than 40%), resulting in convergence adaptation to the arid environment within Central Asia.

On a temporal scale, the community structure of the two deserts may have changed differently. Five series types were identified at the temporal scale in TD based on a long-term (1965–1990; 2012–2013) investigation. Currently, the Artemisia-Krascheninnikovia-Ephemeroids dominate the desert vegetation [32]. Based on the existing data, the constructive species of plant communities have not changed in GD, being mainly *H. ammodendron* or *H. persicum* [29,30]. Thus, the plant community structure in TD could be more sensitive to environmental changes.

### 4.2. Factors Influencing Plant Community Structure in Desert Areas

Based on the stress-dominance hypothesis (SDH), the community assembly mechanism changes from strong abiotic filtration to competition as the environment becomes favorable. However, abiotic filtration will be strengthened, and the species competition level will be increased in arid areas where resources are limited [48]. Therefore, the role of abiotic factors could be as crucial as biotic interaction when analyzing the assembly mechanism of the plant community [49,50]. This study only discussed the impact of abiotic interaction and did not consider the interspecific relationship between the two desert plant communities.

The role of spatial heterogeneity (including climate and soil condition mainly) is vital in plant community structure. There are prominent differences in light, temperature, water, and other factors affecting plant growth and distribution in different habitats [51]. A higher spatial heterogeneity indicates more diverse eco-niches with more coexisting species [10]. Spatial heterogeneity can be on a small scale, e.g., the difference between different slope positions of sand dunes or mountains [27,51]. It can also be large-scale, e.g., environmental heterogeneity between different regions or continents. Longitude and latitude (i.e., geographical factors) can reflect more intense climate, soil, and microhabitat changes [52]. Abiotic factors (geography, climate, and soil) could have a more critical role in community assembly in the extremely arid desert region in Central Asia, whether on a small or a large scale, due to the crucial survivability of plants in the extreme environment [53]. Water conditions can alter the species composition of plant communities and ecosystem functions. This changes the relationship between species diversity and ecosystem multifunctionality [8,54]. The leading factors on the Aral Sea Coast are connected with water management and irrigation. The dynamics of plant communities appear with a series of shifts (successional series)—potamo-, xero-, halo-, and psammosere—characterizing the moisture content and edaphic environments. Therefore, for plant community change, water is the primary limiting factor [55]. For almost all attribute matrices in the present study, the main factors influencing community structure were longitude, climate (MAP, MAT, DSR, and VDP), and soil (SWC, MAST, TN, TP, TK, and TS). Different longitudes may become the main geographical factor, since the study sites had the same latitude, embodying the longitude zonality of desert vegetation in Central Asia [4]. However, the geographical factor interpretation was usually less than that of climate and soil. Thus, the plant community structure based on multiple attributes of the two deserts was relatively similar despite being far apart. TD had higher MAP and SWC than GD (Table 1), closely related to the water vapor supply of Balkhash Lake near the desert and the supplement of the Ili River to groundwater. In contrast, GD had negligible external water supply from lakes or rivers. The overall environmental status in TD was better than in GD, which could be the key reason for the different plant community structures [24,25,56].

Microhabitat diversity or geodiversity (reflecting spatial heterogeneity) also plays a vital role at smaller scales [51,57]. The distribution pattern of herbaceous plants on sand dunes has revealed that they are zonally distributed along the dune with different slope positions. The dune topography is the main influencing factor [27]. In semi-arid areas in Israel, the slopes with high geological and geomorphic diversity seem to buffer the impact of drought years. Thus, they support more diverse plant communities than slopes with low geological and geomorphic diversity. Sand dunes in TD were mostly unstable in trend and diverse in types compared with GD, which was dominated by linear dunes. Thus, it is possible to create rich geological and geomorphic diversity [58], an essential factor influencing the difference in community structure between the two deserts. In addition, the CCA ordination result indicated that the sampling plots in the same desert were scattered, especially in TD (Figure 6). This reflected the geological and geomorphic diversity within a desert. Therefore, we should consider the potential role of high geological and geomorphic diversity regions in mitigating the impacts of climate change on biodiversity when planning conservation and management actions [58].

Soil is also essential to geomorphic diversity, directly controlling the distribution and dynamics of soil moisture in arid areas and affecting plant viability and community development. Soil moisture has a more significant impact on the change in vegetation community structure than soil organic matter, total nitrogen, total phosphorus, and other soil properties in arid ecosystems [59]. However, soil nutrients could be the main limiting factor of plant community distribution patterns in sandy desert grassland [60]. Thus, soil properties, especially soil nutrient availability, are closely associated with SWC, affecting plant distribution and community structure in arid areas [53,61]. SWC, EC, sodium, potassium, calcium, magnesium, chloride, sulfate, pH, organic matter, and gravel contents affect species distribution in the plant communities in the southern part of the Eastern Desert of Egypt [62]. In GD in the present study, the vegetation composition changes with the gradient of SWC, organic matter content, total salt content, sorting coefficient, and pH [26]. TD had significantly higher SWC and SOC, TN, TP, and TK contents than GD (Table 1), jointly affecting plant distribution and community composition (Table S4). The conditions of microclimate, relief, soil tenacity, anthropogenic impact factors, soil transformation by vegetation, ecomorphs of plants, the ratio of the sand fine-grained fractions, and reserves of available moisture are essential in the successional process of natural vegetation in TD [32].

Therefore, for the majority of plant attribute matrices, climatic and soil factors were the primary drivers, with geographic factors playing a secondary role, owing to the spatial heterogeneity (primarily represented by climatic and soil factors) between the two deserts. In recent decades, the surface and land water reserves in Central Asia have changed significantly under the combined effects of climate change and human activities, causing the intensification of the water crisis [63]. The climate in Central Asia is warming, especially in spring. Meanwhile, most of the dry regions across Asia (65.1–99.8%) will face the risk of drought aggravation in the future. Thus, drought intensity and duration are expected to increase [64]. This will further affect the healthy development of the economy, society, and ecosystem in the arid region of Central Asia, threatening plant diversity [3,25]. Therefore, based on the main environmental factors faced by the region, it is necessary to assess the response of plant diversity to future changes for proposing targeted diversity conservation and management measures. Moreover, international cooperation in plant diversity conservation and ecological security should be strengthened among Central Asian countries and regions.

## 5. Conclusions

Depending on multiple plant attributes in two distant temperate deserts with similar latitudes in Central Asia, the differences in community structure were systematically studied. (1) The number of species distributed in different families and functional groups differed obviously between deserts. Similarly, a different distribution pattern was observed among different functional groups between deserts for each of the six plant attributes. (2) The qualitative species similarity was lower than the quantitative one, depicting a relatively strong convergence for plant functional attributes. (3) The overall environmental status in TD was better than in GD. The climatic and soil heterogeneity mainly influenced differences in plant community structure between the deserts. However, the main controlling factors were inconsistent for different plant attribute matrices. (4) We propose that when carrying out international joint biodiversity conservation actions, full attention be paid to the relatively good environmental conditions of TD and the role of GD as a bridge and link in the Central Asian vegetation transition from east to west. This work further deepens our knowledge of the community structure variation in temperate deserts on a large scale and provides valuable insights for regional biodiversity conservation in Central Asia.

## Figures and Tables

**Table 1:** Differences in climate and soil properties between the Taukum Desert (TD) and the Gurbantunggut Desert (GD) in Central Asia.

Item	Desert	MAP (mm)	MAT (°C)	DSR (W m^−2^)	VPD (kPa)	Aridity	SWC (*v*/*v*)	MST (°C)	SOC (g kg^−1^)	TN (g kg^−1^)	TP (g kg^−1^)	TK (g kg^−1^)	TS (%)
Mean	TD	257.0	9.405	15209.3	1.029	0.900	0.534	13.006	1.074	0.209	0.505	21.783	0.392
	GD	163.8	7.879	14982.4	1.192	0.915	0.218	11.222	0.747	0.100	0.294	13.588	0.366
SE	TD	10.5	0.096	15.7	0.007	0.010	0.018	0.037	0.146	0.014	0.044	0.164	0.029
	GD	3.1	0.250	11.3	0.022	0.006	0.014	0.123	0.069	0.008	0.013	0.069	0.008
Sig.		***	***	***	***	ns	***	***	***	***	***	***	ns

MAP: Mean annual precipitation; MAT: Mean annual temperature; DSR: Daily solar radiation; VPD: Vapor pressure deficit; SWC: Soil volumetric water content; MST: Mean soil temperature; SOC: Soil organic content; TN: Soil total nitrogen; TP: Soil total phosphorous; TK: Soil total potassium; TS: Total salt content. ***: *p* < 0.001; ns: *p* > 0.05.

## Data Availability

Not applicable.

## Supplementary Materials

The following supporting information can be downloaded at: https://www.mdpi.com/article/10.3390/plants12183286/s1, Figure S1: Species numbers of different functional groups (life-form, phylogeny, and shrubs with or without assimilative branches (AB)) in plant communities in the Taukum Desert (TD) and Gurbantunggut Desert (GD) in Central Asia; Figure S2: Plant attributes of all species in communities in the Taukum Desert (TD) and Gurbantunggut Desert (GD) in Central Asia. Different lowercase letters indicate significant different between different life-forms at *p* < 0.05; Figure S3: Plant attributes of different families in plant communities in the Taukum Desert and Gurbantunggut Desert in Central Asia; Figure S4: Sørensen (for species only) and Motyka similarity indices of different life-forms based on six plant attributes in plant communities between the Taukum Desert and Gurbantunggut Desert in Central Asia. H: plant height; CD: canopy diameter; PV: plant volume; D: density; TC: total cover; TV: total volume; Figure S5: Sørensen (for species only) and Motyka similarity indices of different phylogenic types (dicotyledons and monocotyledons) and shrubs with (SW) or without simulative branch (SWO) based on six plant attributes in plant communities between the Taukum Desert and Gurbantunggut Desert in Central Asia. H: plant height; CD: canopy diameter; PV: plant volume; D: density; TC: total cover; TV: total volume; Table S1: Correlation coefficients between environmental factors and six plant attributes for all species in communities in the Taukum Desert and Gurbantunggut Desert in Central Asia; Table S2: Correlation coefficients between environmental factors and six plant attributes of different life-forms in plant communities in the Taukum Desert and Gurbantunggut Desert in Central Asia; Table S3: Correlation coefficients between environmental factors and six plant attributes of different phylogenic types in plant communities in the Taukum Desert and Gurbantunggut Desert in Central Asia; Table S4: Correlation coefficients between environmental factors and six plant attributes of shrubs with (SW) and without assimilative branch (SWO) in plant communities in the Taukum Desert and Gurbantunggut Desert in Central Asia; Table S5: Eigenvectors and variance explained between environmental variables and the first two axes of CCA based on six plant attribute matrices in plant communities in the Taukum Desert and Gurbantunggut Desert in Central Asia.
