# Supplementary material for: Combining Multiple Plant Attributes to Reveal Differences in Community Structure in Two Distant Deserts in Central Asia
Source: Plants (Basel). 2023 Sep 17;12(18):3286. doi: 10.3390/plants12183286 (PMC10537988; doi:10.3390/plants12183286)
Supplement: Supplementary file 1 [file plants-12-03286-s001.zip › plants-2591270-supplementary.pdf]

Supplementary materials

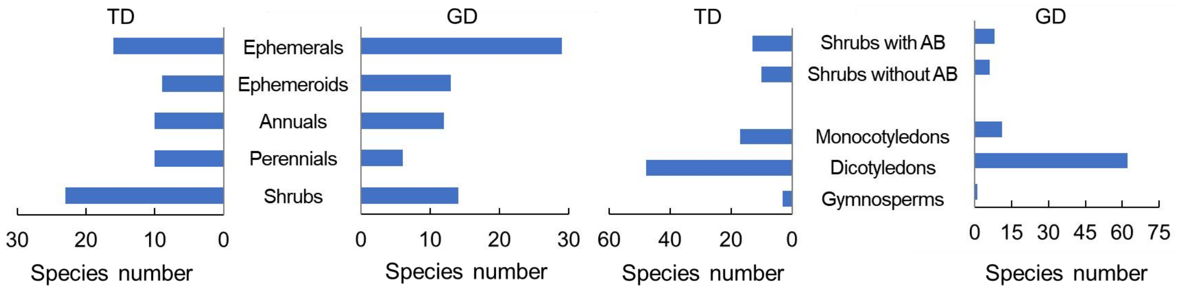

Figure S1. Species numbers of different functional groups (life-form, phylogeny, and shrubs with or without assimilative branches (AB)) in plant communities in the Taukum Desert (TD) and Gurbantunggut Desert (GD) in Central Asia

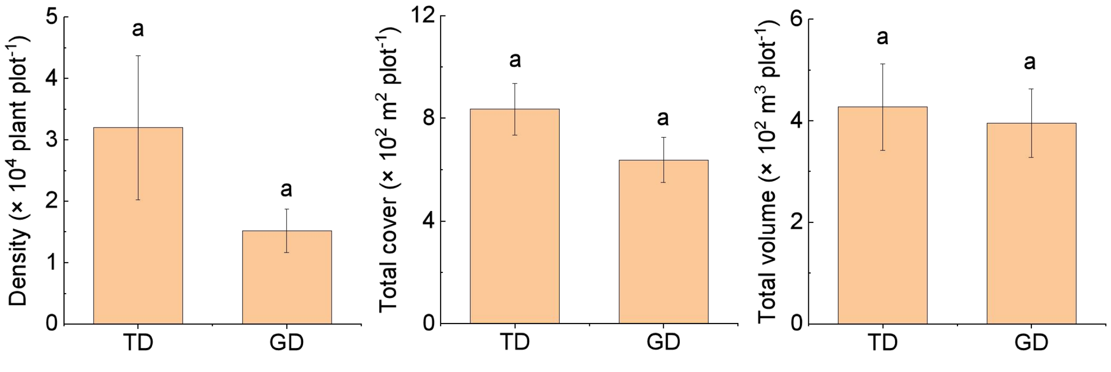

Figure S2. Plant attributes of all species in communities in the Taukum Desert (TD) and Gurbantunggut Desert (GD) in Central Asia. Different lowercase letters indicate significant different between different life-forms at $P< 0.05$.

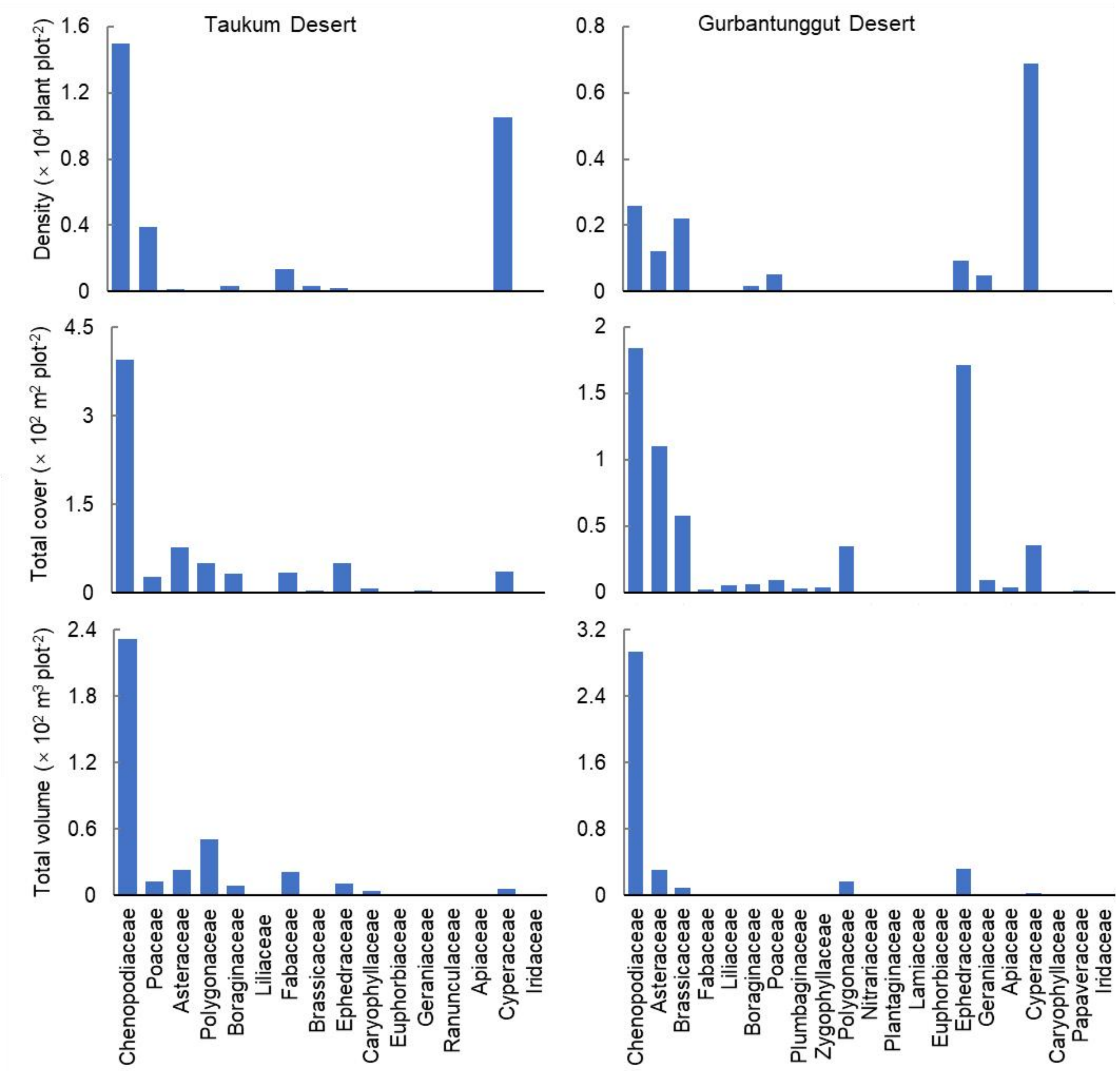

Figure S3. Plant attributes of different families in plant communities in the Taukum Desert and Gurbantunggut Desert in Central Asia

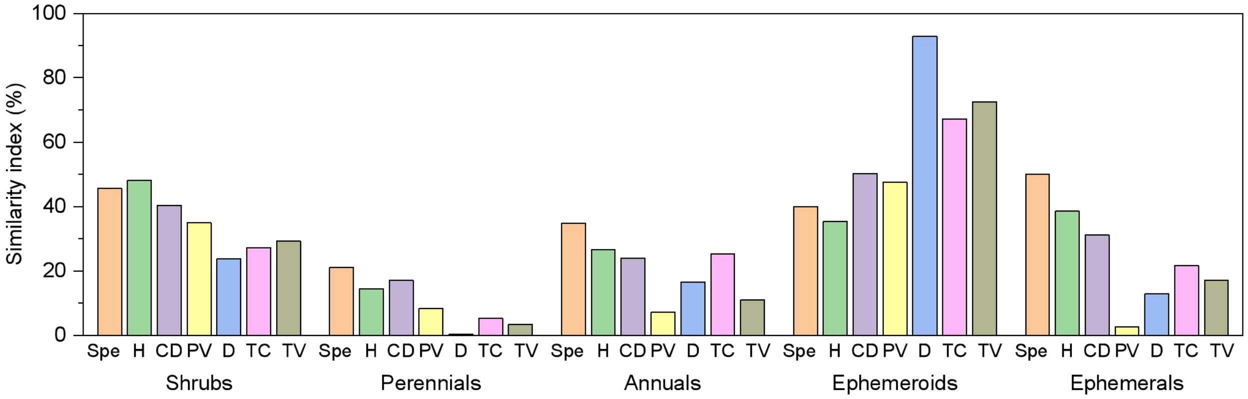

Figure S4. Sørensen (for species only) and Motyka similarity indices of different life-forms based on six plant attributes in plant communities between the Taukum Desert and Gurbantunggut Desert in Central Asia. H: plant height; CD: canopy diameter; PV: plant volume; D: density; TC: total cover; TV: total volume.

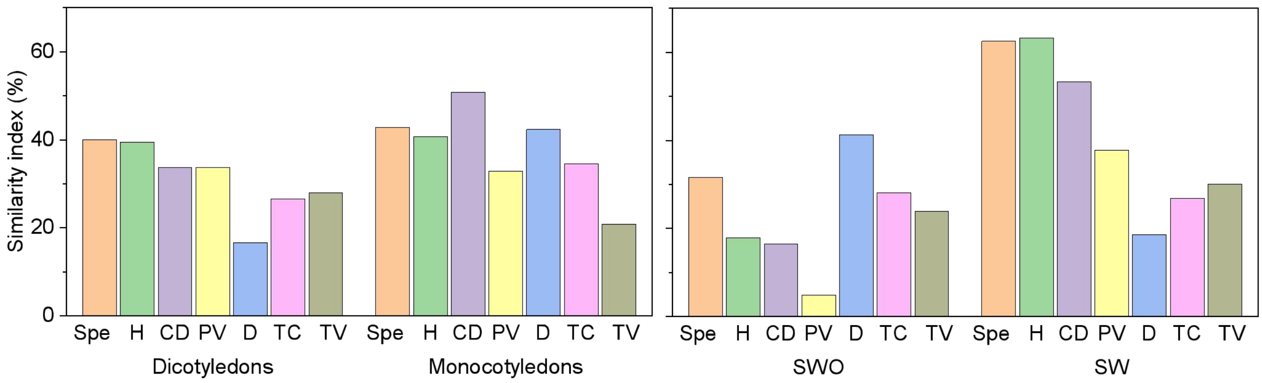

Figure S5. Sørensen (for species only) and Motyka similarity indices of different phylogenic types (dicotyledons and monocotyledons) and shrubs with (SW) or without simulative branch (SWO) based on six plant attributes in plant communities between the Taukum Desert and Gurbantunggut Desert in Central Asia. H: plant height; CD: canopy diameter; PV: plant volume; D: density; TC: total cover; TV: total volume.

Table S1. Correlation coefficients between environmental factors and six plant attributes for all species in communities in the Taukum Desert and Gurbantunggut Desert in Central Asia

Attribute	Longitude	Latitude	Altitude	MAP	MAT	DSR	VPD	SWC	MST	Aridity	SOC	TN	TP	TK	TS
H	−0.695	0.001	−0.380	0.511	0.412	0.564	−0.661	0.622	0.572	−0.091	0.457	0.715	0.253	0.593	0.165
CD	−0.603	0.167	−0.351	0.366	0.304	0.437	−0.466	0.485	0.487	0.010	0.313	0.609	0.104	0.549	0.180
PV	−0.318	0.028	−0.367	0.122	0.049	0.197	−0.343	0.286	0.266	0.138	0.467	0.336	−0.044	0.191	0.056
D	−0.269	−0.159	0.263	0.159	0.280	0.281	−0.332	0.246	0.219	0.111	0.134	0.333	0.449	0.132	−0.003
TC	−0.267	−0.169	0.016	0.178	0.166	0.246	−0.663	0.303	0.128	0.026	0.277	0.421	0.048	−0.091	0.671
TV	−0.078	−0.090	−0.497	−0.067	−0.180	−0.010	−0.268	0.104	0.086	0.068	0.153	0.116	−0.335	−0.048	0.635

H: height; CD: canopy diameter; PV: plant volume; D: density; TC: total cover; TV: total volume. MAP: Mean annual precipitation; MAT: Mean annual temperature; DSR: Daily solar radiation; VPD: Vapor pressure deficit; SWC: Soil volumetric water content; MST: Mean soil temperature; SOC: Soil organic content; TN: Soil total nitrogen; TP: Soil total phosphorous; TK: Soil total potassium; TS: Total salt content. Orange and yellow cells indicated significance at $P < 0.05$ and $P < 0.01$, respectively.

Table S2. Correlation coefficients between environmental factors and six plant attributes of different life-forms in plant communities in the Taukum Desert and Gurbantunggut Desert in Central Asia

Life-form	Attribute	Longitude	Latitude	Altitude	MAP	MAT	DSR	VPD	SWC	MST	Aridity	SOC	TN	TP	TK	TS
Shrubs	D	0.340	0.007	0.561	−0.189	−0.235	−0.299	−0.087	−0.266	−0.487	0.096	−0.242	−0.149	−0.160	−0.564	−0.026
	CD	−0.341	−0.112	−0.421	0.145	0.116	0.263	−0.411	0.293	0.317	0.114	0.531	0.417	0.011	0.184	0.134
	PV	−0.037	−0.054	−0.294	−0.079	−0.172	−0.019	−0.161	0.054	0.039	0.191	0.355	0.094	−0.105	−0.087	0.040
	H	0.148	−0.102	−0.124	−0.150	−0.283	−0.135	−0.219	−0.124	−0.195	0.075	0.353	0.040	−0.224	−0.378	0.168
	TC	−0.053	−0.040	0.032	0.036	−0.107	−0.019	−0.505	0.076	−0.125	0.035	0.046	0.222	−0.279	−0.306	0.470
	TV	0.017	−0.082	−0.487	−0.133	−0.275	−0.099	−0.182	0.016	0.004	0.072	0.124	0.023	−0.386	−0.123	0.579
Perennials	D	−0.219	−0.040	−0.147	0.050	0.062	0.069	−0.154	0.186	0.202	0.146	−0.171	0.459	0.046	0.189	−0.001
	CD	0.171	0.307	0.363	−0.015	−0.219	−0.158	0.203	−0.168	−0.255	−0.108	−0.242	−0.181	0.075	−0.030	−0.117
	PV	−0.092	0.040	0.166	0.202	0.037	0.104	0.023	0.099	0.077	−0.241	0.183	0.051	0.280	0.235	−0.112
	H	−0.233	−0.039	0.166	0.309	0.304	0.282	−0.007	0.263	0.247	−0.098	0.161	0.092	0.487	0.327	−0.389
	TC	−0.340	−0.296	−0.074	0.246	0.097	0.203	−0.319	0.373	0.345	0.046	0.102	0.341	0.298	0.256	−0.209
	TV	−0.449	−0.272	−0.085	0.350	0.249	0.332	−0.344	0.460	0.453	0.008	0.107	0.400	0.387	0.395	−0.220
Annuals	D	−0.282	−0.057	0.176	0.192	0.193	0.288	−0.222	0.256	0.253	0.103	0.233	0.283	0.524	0.245	−0.036
	CD	−0.184	0.404	−0.517	−0.122	−0.013	−0.008	0.103	0.082	0.183	0.175	−0.163	0.225	−0.415	0.367	0.406
	PV	−0.295	0.208	−0.369	0.012	0.090	0.112	−0.098	0.204	0.267	0.205	−0.198	0.380	−0.191	0.347	0.295
	H	−0.577	0.201	−0.611	0.236	0.205	0.372	−0.235	0.421	0.564	0.109	0.238	0.588	0.023	0.691	0.332
	TC	−0.371	−0.003	−0.048	0.255	0.233	0.369	−0.351	0.375	0.326	0.066	0.289	0.364	0.387	0.326	0.425
	TV	−0.431	0.131	−0.276	0.231	0.255	0.358	−0.397	0.393	0.356	0.058	0.248	0.430	0.021	0.393	0.823
Ephemeroids	D	−0.152	−0.038	0.252	0.077	0.328	0.159	−0.320	0.118	0.042	0.024	−0.216	0.141	−0.052	−0.053	0.179
	CD	0.231	0.480	0.421	−0.353	−0.310	−0.302	0.162	−0.282	−0.369	0.400	−0.386	−0.087	−0.177	−0.223	0.113

	PV	−0.045	0.184	0.331	−0.006	0.011	0.058	−0.086	0.019	−0.048	0.167	−0.064	0.117	0.269	0.009	0.030
	H	0.050	−0.024	−0.127	−0.066	−0.036	−0.081	−0.024	0.086	−0.022	−0.005	0.114	0.012	−0.014	−0.130	−0.071
	TC	0.047	−0.097	0.443	0.027	0.140	−0.016	−0.171	−0.038	−0.143	−0.074	−0.233	−0.010	−0.015	−0.224	−0.060
	TV	−0.090	−0.038	0.118	−0.026	0.149	0.023	−0.327	0.085	−0.018	0.086	−0.301	0.166	−0.231	−0.152	0.230
Ephemerals	D	0.246	−0.518	0.141	−0.183	0.007	−0.117	−0.047	−0.172	−0.134	−0.046	0.211	−0.085	0.085	−0.453	−0.160
	CD	−0.519	0.221	−0.288	0.174	0.298	0.373	−0.343	0.439	0.453	0.204	0.356	0.494	0.090	0.476	0.253
	PV	−0.251	0.005	0.136	0.163	0.185	0.249	−0.176	0.212	0.218	0.073	0.197	0.286	0.432	0.232	0.025
	H	−0.562	−0.038	−0.384	0.284	0.355	0.402	−0.362	0.496	0.555	0.069	0.304	0.457	0.109	0.516	0.032
	TC	0.108	−0.331	−0.091	−0.125	0.217	0.028	−0.187	−0.063	−0.044	−0.117	0.251	0.039	−0.141	−0.364	0.027
	TV	−0.153	−0.132	−0.177	0.074	0.353	0.251	−0.324	0.154	0.152	−0.124	0.182	0.204	−0.016	−0.051	0.148

H: height; CD: canopy diameter; PV: plant volume; D: density; TC: total cover; TV: total volume. MAP: Mean annual precipitation; MAT: Mean annual temperature; DSR: Daily solar radiation; VPD: Vapor pressure deficit; SWC: Soil volumetric water content; MST: Mean soil temperature; SOC: Soil organic content; TN: Soil total nitrogen; TP: Soil total phosphorous; TK: Soil total potassium; TS: Total salt content. Orange and yellow cells indicated significance at $P < 0.05$ and $P < 0.01$, respectively.

Table S3. Correlation coefficients between environmental factors and six plant attributes of different phylogenic types in plant communities in the Taukum Desert and Gurbantunggut Desert in Central Asia

Phylogeny	Attribute	Longitude	Latitude	Altitude	MAP	MAT	DSR	VPD	SWC	MST	Aridity	SOC	TN	TP	TK	TS
Gymnosperms	D	0.375	0.028	0.570	−0.224	−0.241	−0.331	−0.024	−0.308	−0.511	0.103	−0.301	−0.189	−0.194	−0.562	−0.040
	CD	−0.055	0.596	−0.138	−0.323	−0.065	−0.151	0.341	−0.081	0.020	0.421	−0.331	−0.096	−0.482	0.279	0.487
	PV	−0.038	0.527	−0.244	−0.294	−0.100	−0.144	0.415	−0.094	0.054	0.337	−0.254	−0.152	−0.416	0.376	0.513
	H	−0.073	0.382	−0.171	−0.176	0.160	−0.040	0.231	−0.010	0.060	0.161	−0.290	−0.082	−0.433	0.260	0.407
	TC	0.360	0.098	0.658	−0.265	−0.176	−0.345	−0.034	−0.320	−0.530	0.166	−0.364	−0.198	−0.298	−0.585	−0.067
	TV	0.351	0.086	0.658	−0.280	−0.122	−0.339	−0.025	−0.321	−0.513	0.171	−0.365	−0.210	−0.337	−0.584	−0.093
Dicotyledons	D	−0.185	−0.144	0.201	0.133	0.171	0.230	−0.194	0.181	0.181	0.068	0.242	0.211	0.482	0.119	−0.054
	CD	−0.700	0.063	−0.333	0.457	0.406	0.535	−0.580	0.607	0.585	0.008	0.348	0.702	0.224	0.621	0.159
	PV	−0.362	0.028	−0.337	0.154	0.083	0.223	−0.379	0.335	0.297	0.158	0.432	0.372	−0.016	0.231	0.055
	H	−0.692	−0.030	−0.317	0.550	0.403	0.570	−0.711	0.629	0.550	−0.135	0.452	0.729	0.282	0.594	0.234
	TC	−0.410	−0.202	−0.348	0.283	0.219	0.396	−0.600	0.427	0.371	−0.063	0.547	0.476	0.181	0.216	0.612
	TV	−0.087	−0.091	−0.549	−0.048	−0.177	0.013	−0.234	0.110	0.118	0.036	0.211	0.108	−0.291	−0.004	0.627
Monocotyledons	D	−0.297	−0.070	0.155	0.118	0.350	0.215	−0.409	0.242	0.184	0.122	−0.217	0.388	0.024	0.100	0.145
	CD	0.129	0.465	0.234	−0.166	−0.150	−0.193	0.315	−0.257	−0.216	0.137	−0.456	−0.170	−0.166	0.061	−0.040
	PV	−0.059	0.127	0.143	0.128	0.024	0.051	0.092	−0.060	0.015	−0.129	−0.213	0.026	0.152	0.196	−0.038
	H	−0.402	0.105	−0.021	0.176	0.362	0.294	−0.232	0.354	0.340	0.217	−0.084	0.321	0.163	0.354	−0.209
	TC	−0.234	−0.102	0.271	0.174	0.229	0.136	−0.313	0.225	0.127	0.048	−0.247	0.285	0.101	0.109	−0.158
	TV	−0.467	−0.097	−0.147	0.232	0.266	0.252	−0.410	0.460	0.397	0.191	−0.176	0.491	0.055	0.365	−0.086

H: height; CD: canopy diameter; PV: plant volume; D: density; TC: total cover; TV: total volume. MAP: Mean annual precipitation; MAT: Mean annual temperature; DSR: Daily solar radiation; VPD: Vapor pressure deficit; SWC: Soil volumetric water content; MST: Mean soil temperature; SOC: Soil organic content; TN: Soil total nitrogen; TP: Soil total phosphorous; TK: Soil total potassium; TS: Total salt content. Orange and yellow cells indicated significance at $P < 0.05$ and $P < 0.01$, respectively.

Table S4. Correlation coefficients between environmental factors and six plant attributes of shrubs with (SW) and without assimilative branch (SWO) in plant communities in the Taukum Desert and Gurbantunggut Desert in Central Asia

Assimilative branch	Attribute	Longitude	Latitude	Altitude	MAP	MAT	DSR	VPD	SWC	MST	Aridity	SOC	TN	TP	TK	TS
SWO	D	−0.416	−0.199	−0.145	0.401	0.130	0.386	−0.628	0.474	0.325	−0.068	0.612	0.438	0.390	0.208	0.128
	CD	−0.630	−0.138	−0.385	0.485	0.402	0.491	−0.508	0.544	0.582	−0.173	0.366	0.606	0.185	0.583	−0.015
	PV	−0.577	−0.139	−0.363	0.447	0.366	0.441	−0.418	0.534	0.556	−0.127	0.409	0.515	0.203	0.537	−0.145
	H	−0.737	−0.250	−0.371	0.629	0.528	0.637	−0.709	0.679	0.663	−0.239	0.403	0.710	0.326	0.691	−0.014
	TC	−0.554	−0.134	−0.356	0.531	0.233	0.480	−0.681	0.523	0.439	−0.238	0.562	0.634	0.262	0.396	0.224
	TV	−0.629	−0.168	−0.445	0.560	0.316	0.535	−0.671	0.567	0.543	−0.257	0.582	0.671	0.244	0.500	0.237
SW	D	−0.026	0.045	−0.118	−0.071	−0.106	0.033	−0.192	−0.009	−0.021	0.131	0.476	0.236	0.014	−0.107	0.106
	CD	−0.444	0.012	−0.305	0.185	0.209	0.340	−0.372	0.378	0.405	0.192	0.485	0.596	0.238	0.354	0.045
	PV	−0.226	0.016	−0.330	0.032	−0.023	0.128	−0.250	0.209	0.204	0.200	0.477	0.338	0.017	0.119	−0.008
	H	0.379	0.028	0.568	−0.227	−0.245	−0.335	−0.021	−0.312	−0.514	0.102	−0.302	−0.193	−0.199	−0.564	−0.037
	TC	0.335	0.049	0.297	−0.340	−0.298	−0.369	−0.122	−0.285	−0.469	0.215	−0.356	−0.188	−0.528	−0.598	0.355
	TV	0.177	−0.044	−0.400	−0.281	−0.369	−0.239	−0.022	−0.127	−0.133	0.141	−0.036	−0.145	−0.468	−0.247	0.556

SWO: shrub without assimilative branches; SW: shrub with assimilative branches; H: height; CD: canopy diameter; PV: plant volume; D: density; TC: total cover; TV: total volume. MAP: Mean annual precipitation; MAT: Mean annual temperature; DSR: Daily solar radiation; VPD: Vapor pressure deficit; SWC: Soil volumetric water content; MST: Mean soil temperature; SOC: Soil organic content; TN: Soil total nitrogen; TP: Soil total phosphorous; TK: Soil total potassium; TS: Total salt content. Orange and yellow cells indicated significance at $P < 0.05$ and $P < 0.01$, respectively.

Table S5. Eigenvectors and variance explained between environmental variables and the first two axes of CCA based on six plant attribute matrices in plant communities in the Taukum Desert and Gurbantunggut Desert in Central Asia

Environmental variable	Height		Canopy diameter		Plant volume		Density		Total cover		Total volume	
	Axis 1	Axis 2	Axis 1	Axis 2	Axis 1	Axis 2	Axis 1	Axis 2	Axis 1	Axis 2	Axis 1	Axis 2
Longitude	-0.904^{***}	−0.105	-0.899^{***}	−0.053	0.466^{*}	0.278	−0.182	−0.359	0.867^{***}	0.061	0.566^{**}	−0.284
Latitude	−0.257	0.286	−0.150	−0.231	−0.245	0.222	−0.166	−0.260	0.018	0.109	−0.142	−0.124
Altitude	−0.164	−0.205	−0.236	0.464^{*}	−0.287	−0.224	−0.022	0.073	0.711^{***}	0.078	−0.152	-0.790^{***}
MAP	0.776^{***}	−0.110	0.724^{***}	0.197	−0.300	−0.354	0.128	0.321	-0.632^{***}	−0.086	-0.418^{*}	0.175
MAT	0.750^{***}	0.075	0.752^{***}	0.192	-0.651^{***}	−0.357	0.100	0.257	-0.522^{**}	0.096	-0.708^{***}	0.019
DSR	0.884^{***}	0.006	0.862^{***}	0.212	-0.553^{**}	-0.382^{*}	0.005	0.348	-0.752^{***}	−0.062	-0.657^{***}	0.140
VPD	-0.809^{***}	−0.173	-0.811^{***}	−0.088	0.388^{*}	0.181	−0.327	-0.443^{*}	0.492^{**}	−0.039	0.457^{*}	−0.105
SWC	0.845^{***}	−0.035	0.764^{***}	0.094	−0.266	-0.397^{*}	0.156	0.330	-0.712^{***}	−0.204	−0.345	0.200
MST	0.812^{***}	−0.011	0.775^{***}	0.052	−0.285	−0.313	0.085	0.306	-0.868^{***}	−0.154	−0.410	0.378
Aridity	−0.080	0.129	−0.086	0.037	−0.242	0.025	−0.099	0.021	0.133	0.018	−0.138	−0.287
SOC	0.228	−0.230	0.168	0.198	0.041	−0.226	−0.360	0.125	−0.280	−0.124	−0.049	0.253
TN	0.732^{***}	0.216	0.786^{***}	−0.095	−0.329	−0.054	0.340	0.600^{***}	-0.681^{***}	−0.064	-0.405^{*}	0.457^{*}
TP	0.764^{***}	-0.527^{**}	0.578^{**}	0.633^{***}	−0.267	-0.715^{***}	-0.712^{***}	0.431^{*}	-0.406^{*}	-0.413^{*}	−0.358	−0.124
TK	0.878^{***}	0.084	0.848^{***}	0.212	-0.473^{*}	−0.293	0.176	0.402^{*}	-0.458^{*}	0.047	-0.539^{**}	0.018
TS	0.599^{***}	0.274	0.633^{***}	0.033	-0.379^{*}	−0.059	0.106	−0.002	−0.156	0.098	−0.407	−0.078
Variance explained (%)	44.3	10.5	42.3	13.3	20.7	19.9	17.8	7.3	17.3	6.2	21.6	20.4

MAP: Mean annual precipitation; MAT: Mean annual temperature; DSR: Daily solar radiation; VPD: Vapor pressure deficit; SWC: Soil volumetric water content; MST: Mean soil temperature; SOC: Soil organic content; TN: Soil total nitrogen; TP: Soil total phosphorous; TK: Soil total potassium; TS: Total salt content. *: $P < 0.05$; **: $P < 0.01$; ***: $P < 0.001$.
